# Supplementary material for: Modeling glioblastoma heterogeneity as a dynamic network of cell states
Source: Mol Syst Biol. 2021 Sep 16;17(9):e10105. doi: 10.15252/msb.202010105 (PMC8444284; doi:10.15252/msb.202010105)
Supplement: Supplementary file 5 — Source Data for Figure 3 [file MSB-17-e10105-s001.zip › Figure3A_sourcedata/GSEA_3065/nosigdb_state1.GseaPreranked.1622718409535/IVY_CT.html]

Details for gene set IVY\_CT[GSEA]

|  || Dataset | state1 |
| Phenotype | NoPhenotypeAvailable |
| Upregulated in class | na\_neg |
| GeneSet | IVY\_CT |
| Enrichment Score (ES) | -0.6679989 |
| Normalized Enrichment Score (NES) | -2.5157156 |
| Nominal p-value | 0.0 |
| FDR q-value | 0.0 |
| FWER p-Value | 0.0 |
Table: GSEA Results Summary

  

Fig 1: Enrichment plot: IVY\_CT      
 Profile of the Running ES Score & Positions of GeneSet Members on the Rank Ordered List

  

| PROBE | GENE SYMBOL | GENE\_TITLE | RANK IN GENE LIST | RANK METRIC SCORE | RUNNING ES | CORE ENRICHMENT || 1 | PTTG1 |  |  | 40 | 0.520 | 0.0132 | No |
| 2 | DBI |  |  | 110 | 0.352 | 0.0179 | No |
| 3 | UBE2C |  |  | 125 | 0.340 | 0.0278 | No |
| 4 | BIRC5 |  |  | 202 | 0.295 | 0.0299 | No |
| 5 | SRPX |  |  | 242 | 0.280 | 0.0352 | No |
| 6 | ATP6V0E2 |  |  | 352 | 0.239 | 0.0320 | No |
| 7 | METTL7B |  |  | 355 | 0.239 | 0.0398 | No |
| 8 | CDC20 |  |  | 469 | 0.212 | 0.0352 | No |
| 9 | AURKA |  |  | 550 | 0.196 | 0.0335 | No |
| 10 | TPX2 |  |  | 556 | 0.194 | 0.0395 | No |
| 11 | CDCA2 |  |  | 859 | 0.150 | 0.0135 | No |
| 12 | CDCA3 |  |  | 994 | 0.134 | 0.0042 | No |
| 13 | KCNIP1 |  |  | 1126 | 0.119 | -0.0053 | No |
| 14 | NMB |  |  | 1149 | 0.118 | -0.0036 | No |
| 15 | FERMT1 |  |  | 1163 | 0.117 | -0.0010 | No |
| 16 | CCNB2 |  |  | 1232 | 0.110 | -0.0044 | No |
| 17 | KIF4A |  |  | 1303 | 0.103 | -0.0081 | No |
| 18 | CKAP2L |  |  | 1359 | 0.099 | -0.0105 | No |
| 19 | CKB |  |  | 1368 | 0.098 | -0.0080 | No |
| 20 | DHFR |  |  | 1413 | 0.094 | -0.0094 | No |
| 21 | EGFR |  |  | 1474 | 0.091 | -0.0125 | No |
| 22 | CENPE |  |  | 1545 | 0.086 | -0.0169 | No |
| 23 | HMGB2 |  |  | 1570 | 0.085 | -0.0165 | No |
| 24 | PBK |  |  | 1651 | 0.079 | -0.0221 | No |
| 25 | NUSAP1 |  |  | 1736 | 0.073 | -0.0283 | No |
| 26 | PRC1 |  |  | 1794 | 0.070 | -0.0318 | No |
| 27 | SAPCD2 |  |  | 1829 | 0.068 | -0.0330 | No |
| 28 | TACC3 |  |  | 1949 | 0.063 | -0.0431 | No |
| 29 | DLGAP5 |  |  | 2028 | 0.058 | -0.0492 | No |
| 30 | SHOX2 |  |  | 2091 | 0.055 | -0.0537 | No |
| 31 | IGF2BP3 |  |  | 2146 | 0.053 | -0.0575 | No |
| 32 | CDCA8 |  |  | 2166 | 0.052 | -0.0577 | No |
| 33 | KIF2C |  |  | 2224 | 0.050 | -0.0619 | No |
| 34 | SMC4 |  |  | 2308 | 0.047 | -0.0688 | No |
| 35 | CENPM |  |  | 2348 | 0.045 | -0.0713 | No |
| 36 | LRRC17 |  |  | 2509 | 0.039 | -0.0864 | No |
| 37 | MYBL2 |  |  | 2549 | 0.038 | -0.0892 | No |
| 38 | GINS2 |  |  | 2567 | 0.038 | -0.0897 | No |
| 39 | DNMT1 |  |  | 2576 | 0.037 | -0.0893 | No |
| 40 | GTSE1 |  |  | 2703 | 0.033 | -0.1011 | No |
| 41 | FBXO5 |  |  | 2870 | 0.028 | -0.1172 | No |
| 42 | BUB1 |  |  | 2888 | 0.028 | -0.1180 | No |
| 43 | ASF1B |  |  | 2929 | 0.027 | -0.1212 | No |
| 44 | OIP5 |  |  | 3357 | 0.017 | -0.1645 | No |
| 45 | CDK1 |  |  | 3396 | 0.016 | -0.1679 | No |
| 46 | HIST1H1D |  |  | 3604 | 0.012 | -0.1888 | No |
| 47 | TTK |  |  | 3724 | 0.009 | -0.2007 | No |
| 48 | NRM |  |  | 3968 | 0.005 | -0.2255 | No |
| 49 | UHRF1 |  |  | 4092 | 0.003 | -0.2380 | No |
| 50 | NCAPG |  |  | 4261 | -0.000 | -0.2553 | No |
| 51 | NUF2 |  |  | 4342 | -0.002 | -0.2634 | No |
| 52 | MELK |  |  | 4558 | -0.006 | -0.2853 | No |
| 53 | COL9A3 |  |  | 4578 | -0.006 | -0.2871 | No |
| 54 | CENPK |  |  | 4697 | -0.007 | -0.2990 | No |
| 55 | SOX21 |  |  | 4829 | -0.010 | -0.3121 | No |
| 56 | CCNE2 |  |  | 5070 | -0.014 | -0.3363 | No |
| 57 | HIST1H1B |  |  | 5125 | -0.014 | -0.3414 | No |
| 58 | BUB1B |  |  | 5187 | -0.015 | -0.3471 | No |
| 59 | KIF14 |  |  | 5262 | -0.016 | -0.3542 | No |
| 60 | CLSPN |  |  | 5848 | -0.026 | -0.4134 | No |
| 61 | PCBP4 |  |  | 6003 | -0.029 | -0.4282 | No |
| 62 | RRM2 |  |  | 6214 | -0.033 | -0.4487 | No |
| 63 | NDC80 |  |  | 6240 | -0.034 | -0.4502 | No |
| 64 | DTL |  |  | 6465 | -0.038 | -0.4719 | No |
| 65 | GAS1 |  |  | 6510 | -0.039 | -0.4751 | No |
| 66 | RNASEH2A |  |  | 6891 | -0.047 | -0.5126 | No |
| 67 | CENPF |  |  | 6902 | -0.047 | -0.5120 | No |
| 68 | LMNB1 |  |  | 7166 | -0.055 | -0.5372 | No |
| 69 | FAM111B |  |  | 7169 | -0.055 | -0.5356 | No |
| 70 | EZH2 |  |  | 7260 | -0.057 | -0.5430 | No |
| 71 | RAMP1 |  |  | 7655 | -0.068 | -0.5812 | No |
| 72 | CDKN2C |  |  | 7660 | -0.068 | -0.5793 | No |
| 73 | TOP2A |  |  | 7678 | -0.069 | -0.5787 | No |
| 74 | LFNG |  |  | 7784 | -0.073 | -0.5871 | No |
| 75 | ASPM |  |  | 7821 | -0.074 | -0.5883 | No |
| 76 | NXPH1 |  |  | 7920 | -0.078 | -0.5958 | No |
| 77 | MSI1 |  |  | 8064 | -0.084 | -0.6077 | No |
| 78 | RAD51AP1 |  |  | 8227 | -0.091 | -0.6213 | No |
| 79 | MCM10 |  |  | 8339 | -0.096 | -0.6295 | No |
| 80 | KIF15 |  |  | 8542 | -0.108 | -0.6466 | No |
| 81 | ESCO2 |  |  | 8566 | -0.109 | -0.6454 | No |
| 82 | TCF12 |  |  | 8614 | -0.112 | -0.6464 | No |
| 83 | BLM |  |  | 8723 | -0.119 | -0.6536 | No |
| 84 | CRISPLD1 |  |  | 8782 | -0.124 | -0.6554 | No |
| 85 | CDCA7 |  |  | 8824 | -0.126 | -0.6554 | No |
| 86 | ID4 |  |  | 8856 | -0.129 | -0.6542 | No |
| 87 | FANCI |  |  | 8917 | -0.136 | -0.6559 | No |
| 88 | FIBIN |  |  | 9036 | -0.145 | -0.6631 | Yes |
| 89 | GATM |  |  | 9049 | -0.147 | -0.6595 | Yes |
| 90 | LRRN1 |  |  | 9054 | -0.147 | -0.6550 | Yes |
| 91 | MEOX2 |  |  | 9060 | -0.148 | -0.6505 | Yes |
| 92 | FADS2 |  |  | 9078 | -0.150 | -0.6473 | Yes |
| 93 | SALL1 |  |  | 9084 | -0.151 | -0.6428 | Yes |
| 94 | KLHDC8A |  |  | 9117 | -0.153 | -0.6409 | Yes |
| 95 | NLGN1 |  |  | 9131 | -0.155 | -0.6371 | Yes |
| 96 | VCAN |  |  | 9151 | -0.158 | -0.6337 | Yes |
| 97 | POU3F2 |  |  | 9173 | -0.162 | -0.6305 | Yes |
| 98 | NPAS3 |  |  | 9181 | -0.163 | -0.6258 | Yes |
| 99 | IGFBP2 |  |  | 9209 | -0.167 | -0.6230 | Yes |
| 100 | KCNQ2 |  |  | 9335 | -0.187 | -0.6296 | Yes |
| 101 | METRN |  |  | 9381 | -0.196 | -0.6276 | Yes |
| 102 | MEX3A |  |  | 9384 | -0.197 | -0.6213 | Yes |
| 103 | SOX8 |  |  | 9456 | -0.215 | -0.6214 | Yes |
| 104 | FREM2 |  |  | 9479 | -0.221 | -0.6162 | Yes |
| 105 | NLGN3 |  |  | 9554 | -0.241 | -0.6158 | Yes |
| 106 | PCDHGC3 |  |  | 9588 | -0.255 | -0.6107 | Yes |
| 107 | CREB5 |  |  | 9616 | -0.265 | -0.6046 | Yes |
| 108 | NES |  |  | 9626 | -0.270 | -0.5965 | Yes |
| 109 | ABAT |  |  | 9646 | -0.278 | -0.5892 | Yes |
| 110 | RFX4 |  |  | 9655 | -0.285 | -0.5805 | Yes |
| 111 | SPATA6 |  |  | 9657 | -0.286 | -0.5711 | Yes |
| 112 | LRRN3 |  |  | 9659 | -0.286 | -0.5616 | Yes |
| 113 | TSC22D4 |  |  | 9677 | -0.296 | -0.5535 | Yes |
| 114 | DLL3 |  |  | 9678 | -0.297 | -0.5436 | Yes |
| 115 | SOX2 |  |  | 9680 | -0.298 | -0.5337 | Yes |
| 116 | BCHE |  |  | 9684 | -0.300 | -0.5240 | Yes |
| 117 | SEMA6A |  |  | 9690 | -0.304 | -0.5143 | Yes |
| 118 | PLP1 |  |  | 9702 | -0.314 | -0.5050 | Yes |
| 119 | HES6 |  |  | 9709 | -0.324 | -0.4948 | Yes |
| 120 | DSEL |  |  | 9714 | -0.327 | -0.4843 | Yes |
| 121 | DCLK2 |  |  | 9717 | -0.328 | -0.4736 | Yes |
| 122 | CSPG5 |  |  | 9721 | -0.330 | -0.4628 | Yes |
| 123 | MLC1 |  |  | 9728 | -0.338 | -0.4522 | Yes |
| 124 | SOX11 |  |  | 9730 | -0.339 | -0.4409 | Yes |
| 125 | SOX9 |  |  | 9754 | -0.358 | -0.4313 | Yes |
| 126 | SDC3 |  |  | 9756 | -0.360 | -0.4194 | Yes |
| 127 | ETV1 |  |  | 9764 | -0.371 | -0.4077 | Yes |
| 128 | OLIG2 |  |  | 9776 | -0.386 | -0.3960 | Yes |
| 129 | ID3 |  |  | 9778 | -0.389 | -0.3831 | Yes |
| 130 | ITGB8 |  |  | 9794 | -0.418 | -0.3707 | Yes |
| 131 | LPL |  |  | 9802 | -0.428 | -0.3571 | Yes |
| 132 | WSCD1 |  |  | 9808 | -0.448 | -0.3427 | Yes |
| 133 | C1orf61 |  |  | 9813 | -0.479 | -0.3271 | Yes |
| 134 | EDNRB |  |  | 9824 | -0.509 | -0.3111 | Yes |
| 135 | DDR1 |  |  | 9829 | -0.524 | -0.2940 | Yes |
| 136 | MATN2 |  |  | 9837 | -0.571 | -0.2756 | Yes |
| 137 | SCD5 |  |  | 9838 | -0.576 | -0.2564 | Yes |
| 138 | OLIG1 |  |  | 9845 | -0.637 | -0.2358 | Yes |
| 139 | FAM181B |  |  | 9854 | -0.697 | -0.2133 | Yes |
| 140 | NOVA1 |  |  | 9858 | -0.741 | -0.1889 | Yes |
| 141 | PTPRZ1 |  |  | 9869 | -0.918 | -0.1593 | Yes |
| 142 | TTYH1 |  |  | 9872 | -1.033 | -0.1250 | Yes |
| 143 | PTN |  |  | 9874 | -1.107 | -0.0881 | Yes |
| 144 | BCAN |  |  | 9878 | -1.175 | -0.0492 | Yes |
| 145 | SCRG1 |  |  | 9879 | -1.473 | -0.0000 | Yes |
Table: GSEA details [plain text format]

  

Fig 2: IVY\_CT: Random ES distribution      
 Gene set null distribution of ES for **IVY\_CT**
